# Marine Invertebrates: Communities at Risk

**DOI:** 10.3390/biology2020832

**Published:** 2013-06-10

**Authors:** Jennifer Mather

**Affiliations:** Psychology Department, University of Lethbridge, 4401 University Drive, Lethbridge, AB, T1K 3M4, Canada; E-Mail: mather@uleth.ca; Tel.: +1-403-329-2423; Fax: +1-403-329-2555

**Keywords:** marine invertebrates, climate change, ecosystem disruption

## Abstract

Our definition of the word ‘animal’ centers on vertebrates, yet 99% of the animals on the planet are invertebrates, about which we know little. In addition, although the Census of Marine Life (COML.org) has recently conducted an extensive audit of marine ecosystems, we still do not understand much about the animals of the seas. Surveys of the best-known ecosystems, in which invertebrate populations often play a key role, show that the invertebrate populations are affected by human impact. Coral animals are the foundation of coral reef systems, which are estimated to contain 30% of the species in the ocean. Physical impact and chemical changes on the water severely damage these reefs, and may lead to the removal of these important habitats. Tiny pteropod molluscs live in huge numbers in the polar seas, and their fragile shells are particularly vulnerable to ocean acidification. Their removal would mean that fishes on which we depend would have a hugely diminished food supply. In the North Sea, warming is leading to replacement of colder water copepods by warmer water species which contain less fat. This is having an effect on the birds which eat them, who enrich the otherwise poor land on which they nest. Conversely, the warming of the water and the loss of top predators such as whales and sharks has led to an explosion of the jumbo squid of the Pacific coast of North America. This is positive in the development of a squid fishery, yet negative because the squid eat fish that have been the mainstay of the fishery along that coast. These examples show how invertebrates are key in the oceans, and what might happen when global changes impact them.

## 1. Introduction

Invertebrates constitute 99% of all the species of animals on the earth, yet they are not what people think of when they hear the word animal. Mammals represent less than 1/10 of one percent of animals, but they are the subject of the focus of animal research and the focus of public attention. Public opinions about invertebrates ranges from dislike and avoidance [1] to ignorance [2], and this second attitude is especially true of marine invertebrates [3]. This is not only biased but dangerous, as invertebrates make up key populations in all the ecosystems of the planet, particularly marine ones [4]. E. O. Wilson, the prominent ecologist, called them 'the little animals that hold up the earth', though not all are little. Regarding the marine ecosystems, we are woefully uninformed about just what animals there are in the oceans, although the Census of Marine Life (COML.org) has generated an exhaustive 10-year effort to identify the major groups in the ocean. They concluded that we have still only identified 10% of the species. 

Understanding the role of invertebrates in marine ecosystems is particularly important because the systems themselves are at risk. The risk comes mainly from human pressure, from physical destruction to overfishing, chemical changes in the seas themselves and human-derived changes in the atmosphere. This includes global warming leading to acidification of the sea [5]. The Census of Marine Life has asked questions through huge widespread surveys, including what global warming will do to oceanic ecosystems, what will happen as a result to biodiversity and how to tell the effects of different pressures we put on the oceans. It is impossible to describe everything that is happening to marine invertebrates and how the ecosystems will respond, so this account will examine the situation of four representative groups: the reef-building corals, the pteropod molluscs, the copepod crustaceans and the Humboldt squid cephalopods.

## 2. Cnidarian Coral Animals

Coral reefs are well known, though it is difficult to realize that the reefs are actually made up of animals. The animals are members of the phylum Cnidaria [6], related to the jellyfish and sea anemones, named because they are predators with stinging cells that can hurt or even kill other animals (think of box jellyfish in Australia). These are fairly primitive animals, without a gut and with a nerve net system that has no centralized brain. However, despite their primitive structures, cnidarians dominate many marine ecosystems. Corals are the only cnidarians that settle on hard surfaces, grow clones of themselves on one another and form the structural basis for ecosystems. They are primarily found in warm, clear tropical waters, which led scientists to wonder what their energy source was, as there were no tiny plants or animals to eat. Corals are symbiotic, closely associated with algae that actually lived inside their tissues. The algae supply the corals with nutrients and the corals in turn supply the algae with carbon dioxide to fuel their photosynthesis, allowing them to thrive in an otherwise marginally inhabitable region.

Corals are not just important to the ecosystem in which they are found, they are its foundation. The coral reefs are often compared to the tropical rainforests on land for diversity of life and productivity. They are the most diverse marine habitat per unit area [7], although they only make up about 0.1% of the earth's surface and 0.2% of the ocean’s. There are probably only about 1,000 coral species, but the structures that these corals build support for one third of all the animal species found in the oceans. Many of these animals (molluscs, crustaceans, worms, echinoderms, cnidaria—and fish) are tiny, and there appears to be a high rate of endemism (found in only one small area). That means if a single reef is destroyed, a whole array of species it supports is wiped out—they cannot be found anywhere else.

This rich array of animal life is at risk because of a host of things that humans have been doing to reefs, both locally and globally, for years [8]. Locally, we degrade reefs by poor water quality, including poisonous runoffs from the land (see the example of Florida), by physical damage to the reefs by such impacts as anchors and dredges. Collectors of tropical fish in the Indo-east Pacific were even known to dynamite reefs to “get at” the little fish inside them. We also degrade reef systems locally by overfishing; in much of the Caribbean large fishes are essentially absent, taking key species out of the web of the ecosystem. Sometimes we damage coral reef ecosystems by importing invasive species—a species of algae escaped from the aquarium at Monte Carlo and has invaded much of the Mediterranean, and lionfish from Indonesia are disrupting the ecosystems of the Caribbean and North Atlantic.

Global threats to coral reefs are equally destructive. The release of carbon dioxide into the atmosphere due to human activities, mostly burning fossil fuels but also deforestation and industrialization, is easy to document. This increase of CO_2_ is 100 times faster over the last 250 years than it has been over the last 650,000 [9]. Half of the carbon dioxide released into the air is taken up by the waters of the oceans, and over this short period the pH of the oceans has dropped by exponential 0.1 units. Ocean acidification does not sound like a problem until we realize that many ocean organisms, like the corals, absorb calcium from the ocean to build their skeletons. It has been suggested that one third of all corals are at risk of extinction [8]. As the CO_2_ levels rise, coral may lose 20–60% of their calcification. Reef building balances erosion, and weaker reefs will erode quickly. Global warming is equally destructive of ecosystems, though perhaps not so much for coral ones [9]. Probably the biggest risk is that local and global influences may combine to really disrupt the ecosystems. There is a principle that adds to this, that when we disrupt an ecosystem, it may be very difficult to restore it. When corals die, the surfaces are covered with algae, and new coral animals need hard surfaces, now unavailable, on which to settle.

Scientists and conservationists, and even the general public, know that the coral reefs are at risk, although we know so little about these ecosystems that we may not know what to do about it. Decreasing the CO_2_ in the atmosphere, would help the corals but the Kyoto agreement, weak as it is, is not even being followed [10]. Scientists want to study reefs with different amounts of disruption, though they now find it difficult to locate non-degraded ones [8]. One partial solution is to designate Marine Protected Areas (MPAs) [11], and Australia has led the way with protection of the Great Barrier Reef system. However, this is only a small part of the reef ecosystems in the world, and most others are at risk of local changes that could kill them off. Ironically, scientists do not know how much disruption a coral reef system can take, and even find it difficult to locate 'pristine' reefs that have not been damaged by local influences [8]. 

## 3. Shelled Pteropod Molluscs

Not all invertebrates dominate an ecosystem, some are only a key part of the web. The story of the threat to thecosomatous pteropods is set in the open-ocean drifting plankton, particularly polar waters. However, first, what is a pteropod? It is a mollusc, not heavy-shelled like other snails but with a light aragonite shell, that does not drag it down into the ocean deep. Pteropods are tiny (<1 cm across), but hugely abundant seasonally in summer in the cold waters of the polar areas [12]. They are the second step up in the food chain, eating phytoplankton (the tiny plants that are the primary producers in most of the ocean). They are then eaten by larger planktonic animals, but also by fish, some seabirds and even whales (think of the filter-feeding baleen whales). While tiny, they are not simple animals. They catch the phytoplankton by filter feeding or by making mucus webs, leaving them out to catch planktonic plants and then eating food and webs. As phytoplankton migrate daily, up to the surface to photosynthesize in the day and down deeper at night (probably to try and escape predators), the pteropods follow them. In the northern polar waters there is only one species, *Limacina helicena*, which has a one-year life cycle. As the food supply is seasonal in the high Arctic, many species like the pteropods reproduce in summer, live through the winter as juveniles, probably reducing their metabolism [13]. It is a challenging ecosystem and a difficult life.

The reason that pteropods are the target of concern is that their thin, fragile shells are made up of aragonite [9], a form of calcium carbonate which is most soluble in water. When they die, their shells 'import' calcium to the deep ocean where other animals can utilize it [12]. However, this aragonite deposition is threatened by the increased acidification of the water, and CO_2_ is most readily absorbed by cold polar water. Models of the effect of ocean acidification point to the surface water of the polar oceans as most vulnerable to ocean acidification. If ocean acidification means that these waters are less than saturated with aragonite, then pteropods will either restrict their range to deeper in the water, or move into corrosive water that could eat away their skeleton. Alternately, they may be forced to move to less saturated warmer water, but many polar sea animals have special adaptations to the cold and cannot tolerate warmer water [9]. 

Research on acidification effects on pteropod skeletons is quite recent. However, cultured pteropods kept at the acidification expected for 2100, given the rate of CO_2_ increase in the atmosphere, would lose 28% of their aragonite, a significant amount. Another study evaluated risk on the basis that juvenile and developmental stages are always a higher risk for damage from environmental variations than adults (think of the risk of the prenatal period for humans), including the cold of the polar winter. They found that elevated temperature before winter was the larger contributor to juvenile mortality, although acidification also mattered [13]. In other words, the effects of climate change on pteropods is dual and may be different at different times of their life cycle. In addition they may adapt in a variety of ways. As one reference says “a decline of their populations would likely cause dramatic changes to the structure, function and services of polar ecosystems” [12].

It is difficult to know what we could do to mitigate these effects. We can place coral reefs in Marine Protected areas, we can stop sewage outflow to the bays and minimize structural damage. We cannot do that for Pteropods. Huge areas of the ocean, with millions of these tiny animals, cannot be affected by small local changes. The only thing we can do to minimize the damage we have already caused to the animals in ocean ecosystems that depend on calcium for shells, bones or support is to stop emitting such volumes of greenhouse gases. Ironically, the general public is alerted to the threat of global warming of Arctic ecosystems by the possible decline of polar bears [14]. This is a classic example of “charismatic megafauna”, the attraction of large beautiful mammalian species to us. Yet the risk to the polar ecosystems and ultimately to life on earth is much larger from the degradation of marine animal skeletons.

## 4. Copepod Crustaceans

While pteropods may produce large numbers and dominate local ecosystems, copepods are one of the most common group of animals on earth and probably the most important herbivores in the ocean. They are really tiny, 1–2 mm long, and they eat the even tinier phytoplankton. They have the exoskeleton of the crustaceans, a front and center eye and the pair of large second antennae that can move them through the water with a kind of rowing motion [6]. They are an absolutely critical second stage in the marine ecosystems of the open ocean, fed on by fishes including economically important species, other invertebrates and even some filter-feeding mammals. Because they are so tiny their speciation is not well known but they seem to be not as globally diverse as other animal groups, yet quite diverse locally [12]. As well as dominating their level of the food chain, copepods return nutrients to the ocean waters when they die and drift into the deeps.

Different *Calanus* copepod species dominate in waters of different temperatures, and that can make a difference to the higher levels of the food chain in surprising ways. Oceans have not been monitored over all areas or for very long. However, a study by the Sir Alistair Hardy Foundation for Ocean Science has monitored both water temperature and copepod population over 40 years, 1960–1999 in the North Atlantic [15]. Warm water from the south has begun to invade the eastern North Atlantic and the North Sea, and along with it have come more warm-water copepods and fewer cold-water ones. Interestingly, in the northwestern North Atlantic there are more cold water species, and the temperature there is getting colder because of the North Atlantic oscillation in the Labrador Sea. Since the North Sea supports an intensive fishery, changes in temperature may affect the fishery directly by decreasing the population of cod, which is already at risk because of overfishing, and may be impacted more by the change in food species for them.

There is another long-distance and long-term effect on the North Atlantic ecosystems. It turns out that cold-water Arctic copepods are not only larger but also richer in fats than warmer water ones. This can have consequences for not only the fish that eat them but also predatory birds [16]. Little auks eat mainly copepods and nest in widespread colonies in the interior of arctic islands. Fish eating birds such as guillemots eat from higher up the food chain, and nest on coastal cliffs. With fewer fat-rich copepods, the auks may face starvation or at least a major energy input decrease because they have to go farther to find appropriate food. So their breeding will be disrupted. Meanwhile the guillemots are doing fine. However, the Arctic land ecosystems are nutrient-poor. An auk colony makes a significant contribution to the local energy budget from its guano, and from protein from other organic matter. This is released slowly, fuels plant growth and fosters a more diverse ecosystem around these 'hot spots'. More dense and diverse vegetation can allow other species of animal to invade, and a more complex ecosystem can provide food for scavengers and predators. In other words, they enrich the land ecosystems. In contrast, the guillemots nest on rocky cliffs and excrete guano directly into the sea or where it is washed into the sea after the next (frequent) storm. 

Of course the opposite could be happening in the western North Atlantic, where the sea temperature is falling, but no one is studying this situation there. The problem illustrates a fact known to ecologists that we must not ignore, that an ecosystem is like a house of cards. Pull out one piece, as in the overfishing of cod on the Grand Banks of eastern North America, and many aspects of the system will be changed. Other species have invaded the Grand Banks, including invertebrates and fish species such as Arctic cod that do not grow to the size of the Atlantic cod formerly found there [17]. When we disrupt ecosystems they may not return to their former structures.

## 5. Humboldt (Jumbo) Squid

Not all marine invertebrates are tiny inhabitants of the lower levels of the food chain. Cephalopods can range from a few cm long to giant squid with a length, not including the tentacles, of over 12 meters. They are really different offshoots of the molluscs [18], having lost the molluscan protective shell. In addition, probably in competition with the bony fishes, coleoid cephalopods have a centralized brain, high metabolic rate, fast speed, many flexible arms and the best camouflage in the animal kingdom. They inhabit the middle ranges of ecological webs, consuming molluscs, fish and crustaceans and being preyed on in turn by larger fishes and marine mammals. In contrast to the fishes’ slow development to maturity, the cephalopods live only 1–2 years, attaining their size by a very efficient metabolic turnover of food into tissue and a voracious appetite. Thus jumbo squid are perhaps poised to invade ecosystems, having fast growth, tolerance of a wide range of temperatures [19] and high intelligence. No one has studied their intelligence, as they are difficult to keep in captivity as they have a body length of 2 meters, fast metabolism and jet propulsion to escape from capture. However, their octopus and cuttlefish relatives are known to be intelligent [20]. Jumbo squid have received the reputation of being 'aggressive', and this reputation may stem from divers and fishers who go into the water when these large animals (also known to be cannibalistic) are in a feeding frenzy. Still, they are equipped with a parrot-like bill and rows of suckers with hooks on them, so they can retaliate.

Humboldt squid (*Dosidicus gigas*) are mostly tropical, living along the Pacific coasts of the Americas and into the Gulf of California in particular. Being at mid-trophic levels, they consume anchovy off the South American coast and sardines and mackerels in the Gulf of California. They are eaten in turn by top predators such as large sharks, tuna, sea lions, dolphins and sperm whales. They feed at night, and their vertical migrations may be tracked in the Gulf of California by sperm whales [21]. However, this list of their predators represents oceanic species in peril, and as the population of their predators declined, the populations of the jumbo squid have increased [19]. Jumbo squid are now a major part of the ecosystem in the Gulf of California. Since 1995, there has been a thriving jumbo squid fishery in Baja California, around 100,000 tons/year. We must hope they are not going to be over-exploited by the fishers in turn.

But something else interesting is happening to the jumbo squid that suggests warming of the waters as well as the lack of top predators is having an effect on the Pacific coast ecosystems [22]. Jumbo squid began to be found off Monterey, and then up the coast to eventually be seen in Puget Sound and off Vancouver Island. El Nino ocean current events may have allowed the jumbo squid to move up the California coast, along with an increase in the picoplankton that are food for their parlarvae. However, in Monterey Bay, the population of Pacific hake fish decreased as the jumbo squid population increased, and the hake is the most abundant commercial fish species on the western coast of the US and Canada. It is rare to see an invasive species of such size and so high in the ecosystem levels, but that does appear to be what is happening with the jumbo squid. It may not be the warming of the oceans alone, or the decimation of their former predators. Jumbo squid are quite tolerant of temperature variation and, like all cephalopods, they are able to shift their diet to take advantage of local abundance and have the fast turnover of a short lifespan to take advantage of such opportunities. Perhaps these invertebrates will come to dominate marine ecosystems that have been depleted of fish by climate change, overfishing and selective catching. Perhaps this is an advantage for a fishery that harvests such an abundant source of protein. We shall see, but it reminds us again that tampering with an ecosystem has consequences far wider than we suspected.

## 6. Conclusions

What do these examples demonstrate about the disruption of ecosystems by damage to invertebrate populations? The reasons for these four examples are clear—ocean acidification, temperature warming and overfishing. The changes are not inevitable but mostly the result of human impact, especially the release of greenhouse gases. Although global warming is understood by most people, it is easy despite this to take the short-term economically fueled solution and do nothing much. Pressure on policy makers, more economic use of the resources of the planet by actions such as reduction of emissions, recycling of waste and consumption of less meat will all reduce our impact on corals, pteropods, copepods and squid, as well as many other important animals. The alternative is major reorganizations of the invertebrate-based ocean ecosystems like those shown here that support us, many of which will be inconvenient and a few of which might be catastrophic. 
